# Predicting Severe Respiratory Failure in Patients with COVID-19: A Machine Learning Approach

**DOI:** 10.3390/jcm13237386

**Published:** 2024-12-04

**Authors:** Bahadır Ceylan, Oktay Olmuşçelik, Banu Karaalioğlu, Şule Ceylan, Meyha Şahin, Selda Aydın, Ezgi Yılmaz, Rıdvan Dumlu, Mahir Kapmaz, Yeliz Çiçek, Abdullah Kansu, Mustafa Düger, Ali Mert

**Affiliations:** 1 Department of Infectious Diseases and Clinical Microbiology, Medical Faculty, Istanbul Medipol University, Istanbul 34214, Türkyie; meyha.sahin@medipol.edu.tr (M.Ş.); seldaaydin@medipol.edu.tr (S.A.); ezgi.yilmaz@medipol.edu.tr (E.Y.); ridvan.dumlu@medipol.edu.tr (R.D.); mahir.kapmaz@medipol.com.tr (M.K.); yeliz.cicek@medipol.edu.tr (Y.Ç.); 2 Department of Internal Medicine, Medical Faculty, Istanbul Medipol University, Istanbul 34214, Türkyie; oolmuscelik@medipol.edu.tr (O.O.); alimert@medipol.edu.tr (A.M.); 3 Department of Radiology, Medical Faculty, Istanbul Medipol University, Istanbul 34214, Türkyie; banu.karaalioglu@medipol.edu.tr; 4 Department of Nuclear Medicine, University of Health Science, Gaziosmanpaşa Training ve Research Hospital, Istanbul 34668, Türkyie; suleceylan2003@yahoo.com; 5 Department of Chest Diseases, Medical Faculty, Istanbul Medipol University, Istanbul 34214, Türkyie; akansu@medipol.edu.tr (A.K.); mduger@medipol.edu.tr (M.D.)

**Keywords:** COVID-19, machine learning, prognosis

## Abstract

**Background/Objectives:** Studies attempting to predict the development of severe respiratory failure in patients with a COVID-19 infection using machine learning algorithms have yielded different results due to differences in variable selection. We aimed to predict the development of severe respiratory failure, defined as the need for high-flow oxygen support, continuous positive airway pressure, or mechanical ventilation, in patients with COVID-19, using machine learning algorithms to identify the most important variables in achieving this prediction. **Methods:** This retrospective, cross-sectional study included COVID-19 patients with mild respiratory failure (mostly receiving oxygen through a mask or nasal cannula). We used XGBoost, support vector machines, multi-layer perceptron, k-nearest neighbor, random forests, decision trees, logistic regression, and naïve Bayes methods to accurately predict severe respiratory failure in these patients. **Results:** A total of 320 patients (62.1% male; average age, 54.67 ± 15.82 years) were included in this study. During the follow-ups of these cases, 114 patients (35.6%) required high-level oxygen support, 67 (20.9%) required intensive care unit admission, and 43 (13.4%) died. The machine learning algorithms with the highest accuracy values were XGBoost, support vector machines, k-nearest neighbor, logistic regression, and multi-layer perceptron (0.7395, 0.7395, 0.7291, 0.7187, and 0.75, respectively). The method that obtained the highest ROC-AUC value was logistic regression (ROC-AUC = 0.7274). The best predictors of severe respiratory failure were a low lymphocyte count, a high computed tomography score in the right and left upper lung zones, an elevated neutrophil count, a small decrease in CRP levels on the third day of admission, a high Charlson comorbidity index score, and a high serum procalcitonin level. **Conclusions:** The development of severe respiratory failure in patients with COVID-19 could be successfully predicted using machine learning methods, especially logistic regression, and the best predictors of severe respiratory failure were the lymphocyte count and the degree of upper lung zone involvement.

## 1. Introduction

COVID-19 manifests in diverse clinical courses, ranging from asymptomatic or mild cases to severe respiratory failure and death for some individuals [1]. Severe respiratory failure is defined as the need for high-flow oxygen support, continuous positive airway pressure (CPAP), mechanical ventilation, and extracorporeal membrane oxygenation (ECMO). Some therapies are available for patients experiencing this serious condition. Notably, dexamethasone treatment has been shown to reduce mortality among COVID-19 patients requiring oxygen therapy [2]. Additionally, tocilizumab has been recommended alongside steroid treatment for patients requiring high-flow oxygen, CPAP, or mechanical ventilation, with early administration proving more effective [3].

Numerous studies have employed machine learning methods to forecast the deterioration of respiratory function in individuals with a COVID-19 infection [4,5,6,7,8,9,10,11,12,13,14,15,16,17,18,19,20,21,22,23,24,25]. These studies exhibited varying degrees of success in predicting their endpoints. The differences in predictive success among these studies stem from several factors, including the severity of the patient’s illnesses, the target variables, the independent variables used for estimation, and the sample sizes. Each study focused on different patient populations with varying disease severity, which may have led to the differences in predictive accuracy. Additionally, variations in the selection and inclusion of independent variables could have influenced the model’s predictive power. Lastly, differences in sample sizes might have affected the robustness and generalizability of the predictive models developed in each study.

These algorithms have enabled healthcare facilities to prioritize patients with poorer prognostic factors, thus preventing healthcare system overload and facilitating close monitoring and early intervention for at-risk patients.

Although there are many studies in the literature that aim to predict respiratory deterioration in COVID-19 infections, few studies have tried to achieve this goal by combining demographic characteristics, clinical findings, laboratory values, and radiological scoring. In our study, we employed machine learning methods to predict the progression to advanced respiratory failure among patients hospitalized with mild respiratory failure, utilizing easily accessible demographic, clinical, radiological, and laboratory data. While limited, the existing literature suggests that the severity of COVID-19 may vary based on the involvement of specific lung lobes, with upper lobe involvement potentially indicating more serious respiratory failure [26,27,28]. Considering this, we included data on lung lobe involvement from computed tomography (CT) scans in our study when examining variables affecting the progression to advanced respiratory failure. Furthermore, evidence suggests that elevated serum CRP levels, procalcitonin levels, and neutrophil counts, along with decreased lymphocyte counts, are associated with severe COVID-19 outcomes [29,30,31,32,33,34,35,36,37]. However, few studies have explored the impact of changes in these variables over time on the development of respiratory failure. Our study included COVID-19 patients hospitalized with mild respiratory failure, all of whom received steroid treatment, and we assessed changes in serum CRP, procalcitonin, neutrophil, and lymphocyte values on the third day of treatment as potential predictors of worsening respiratory failure. Our objective was to predict the progression to severe respiratory failure among hospitalized COVID-19 patients with mild respiratory failure using machine learning models. Additionally, we sought to identify the most valuable independent variables for predicting the development of severe respiratory failure.

## 2. Materials and Methods

This was a retrospective, cross-sectional study focusing on COVID-19 patients with mild respiratory failure admitted to and monitored at Istanbul Medipol University Hospital between January 2020 and January 2022. The study was approved by the institutional review board and local ethics committee of Istanbul Medipol University.

‘Mild respiratory failure’ was defined as a resting respiratory rate of ≥22/minute, a resting oxygen saturation level < 94%, or experiencing decreased saturation with minimal exertion. These cases were monitored in the ward to see if they developed severe respiratory failure. ‘Severe respiratory failure’ was assigned based on the use of CPAP or high-flow oxygen support to meet a patient’s oxygen needs. In all patients with a COVID-19 infection whose respiratory functions deteriorated, high-flow oxygen and CPAP treatments were applied before mechanical ventilation or ECMO was applied. Therefore, only patients who received high-flow oxygen and CPAP treatment were considered as patients with severe respiratory failure. Cases were divided into two groups: those who developed severe respiratory failure and those who did not during follow-ups.

**Inclusion Criteria:**

Confirmed positive SARS-CoV-2 PCR based on a nasopharyngeal swab taken within three weeks before hospital admission;

Age over 18 years;

Hospitalization and treatment on the ward between January 2020 and January 2022 due to COVID-19;

Mild respiratory failure.

**Exclusion Criteria:**

Patients receiving high-flow oxygen therapy, CPAP, mechanical ventilation, or ECMO therapy at first presentation;

Patients admitted to intensive care at first presentation;

Patients with concurrent conditions such as pulmonary edema or pulmonary embolism unrelated to COVID-19 that may have contributed to respiratory failure;

Patients without respiratory failure at first presentation.

**Variables Evaluated in This Study**

We collected and analyzed the following data from patient records:

**Demographic information:** Age, gender, and body mass index (BMI).

**Clinical history:** Mode of transmission (domestic, outdoor, or unknown), smoking history (including the number of cigarettes smoked), time from symptom onset to dyspnea onset and hospital admission, and symptoms at disease onset (cough, weakness, fever, body pain, sore throat, and diarrhea).

**Comorbidities:** Underlying diseases (liver transplantation, cancer with or without metastases, heart failure, leukemia, myasthenia gravis, lymphoma, rheumatoid arthritis, aplastic anemia, multiple myeloma, cerebrovascular diseases, aortic aneurysm, dementia, Parkinson’s disease, hypertension, ischemic heart disease, diabetes mellitus with or without end-organ damage, chronic obstructive pulmonary disease, bronchiectasis, asthma, idiopathic pulmonary fibrosis, severe or mild chronic renal failure, and renal transplantation), pregnancy, immunosuppression, angiotensin-converting enzyme inhibitor usage, and previous steroid usage. Charlson comorbidity index values were calculated using patient information [38]. Severe renal failure was defined as receiving a renal transplant and a having creatinine level greater than 3 mg/dl. Patients with renal failure who did not have a renal transplant and whose creatinine level was not higher than 3 mg/dl were defined as having mild renal failure. Patients were considered to have diabetes with end-organ damage if they had diabetic retinopathy, diabetic nephropathy, or diabetic neuropathy.

**Laboratory parameters:** Serum D-dimer and ferritin levels at upon first hospital admission. Neutrophil, leukocyte, and lymphocyte counts, and serum C-reactive protein (CRP) and procalcitonin levels at upon first hospital admission and on the third day of hospitalization.

**Treatments and interventions employed due to COVID-19:** High-flow oxygen support, CPAP, intensive care unit admission, use of steroids, and the use of specific medications (e.g., remdesivir or favipiravir).

**Imaging findings:** Total and individual lung zone computerized tomography (CT) scores [39].

In our study, all cases received steroid treatment, and some were administered anakinra and tocilizumab during follow-up. However, these treatments were not considered to be variables affecting the outcome in our analysis. The severity of the patient’s illnesses varied during their hospital stay, and these treatments were adjusted accordingly, making it difficult to assess the effects of medications on the development of severe respiratory failure. Nevertheless, antiviral treatments were included among the variables considered to affect the worsening of respiratory failure, as they were administered consistently regardless of disease severity.

**Statistical analysis:**

**A. General group comparisons:**

The patient groups with and without severe respiratory failure during the follow-ups were compared in terms of the variables listed above. SPSS 17 software was utilized for general group comparisons. Categorical variables, normally distributed continuous variables, and non-normally distributed continuous variables were reported as percentages, means ± standard deviations, and medians (ranges), respectively. Student’s t-tests and the Mann–Whitney U-tests were used to compare normally distributed and non-normally distributed variables between groups, respectively. The chi-squared test was employed for the comparison of categorical variables. A *p*-value of <0.05 was considered statistically significant.

**B. Machine learning approach:**

**Figure 1:**
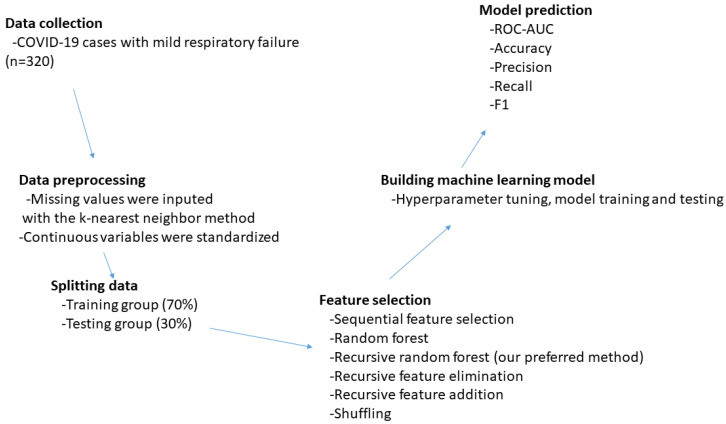
Steps of the machine learning approach used to predict the development of severe respiratory failure among COVID-19 patients.

Various machine learning methods were tested to predict the development of severe respiratory failure during follow-ups. The steps of the machine learning approach used to predict the development of severe respiratory failure among COVID-19 patients are summarized in Figure 1.

**1. Data preprocessing:**

Categorical variables were encoded as dummy variables, while continuous variables were standardized. There were missing values regarding symptom duration in 41 cases; computed tomography scores in 22 cases; leukocyte, neutrophil, and lymphocyte counts in 11 cases; serum CRP levels in 22 cases; and procalcitonin levels on the third day of the follow-up in 151 cases. These missing values were input using the k-nearest neighbor method. The dataset was divided into 2 groups, 70% training and 30% testing, for the application of machine learning methods.

**2. Feature selection:**

To reduce the number of independent variables and eliminate unnecessary ones, the following methods were employed.

a. A filtering method: Correlated, constant, quasi-constant, and duplicate data were removed using the feature engine program, with thresholds set at 80%, 0.998, and the default settings, respectively.

b. Recursive feature selection: This embedded method, utilizing random forest, further reduced the number of variables.

By applying the two methods noted above, the variables included at the beginning of this study were reduced to the following:

Age;

Lymphocyte count;

Neutrophil count;

Ferritin;

Symptom duration;

CT scores of individual lung zones (right upper, right lower, left upper, and left lower lobes);

Dyspnea onset time;

CRP; Procalcitonin;

D-dimer;

Charlson comorbidity index;

Body mass index;

Decrease in leukocyte count on the third day;

Decrease in lymphocyte count on the third day;

Decrease in serum CRP level on the third day;

Decrease in serum procalcitonin level on the third day;

Mode of transmission.

Using these variables, machine learning models were developed to predict the progression to severe respiratory failure in COVID-19 pneumonia cases with mild oxygen requirements.

**3. Model development, evaluation, and explainability:**

Machine learning methods were applied, with the help of a Python program, to predict the development of severe respiratory failure among COVID-19 patients with mild respiratory failure.

Binary classification models were developed using various algorithms, including XGBoost, radial basis function support vector machines (SVMs), multi-layer perceptron (MLP), k-nearest neighbor (KNN), random forests (RFs), decision tree, logistic regression, and **naïve Bayes**.

Ensemble models were also developed, using these algorithms for each feature set. Hyperparameters were optimized using GridSearchCV.

Models were evaluated and compared based on various metrics, including accuracy, area under the receiver operating characteristics curve (AUC ROC), precision, recall, and F1 score. These metrics provide insights into the performance of each model and help in selecting the best-performing model for the task at hand. These metrics can be easily calculated with the help of the confusion matrix, as shown below.

**True Class****Positive****Negative****Predicted class****Positive**True positiveFalse positive**Negative**False negativeTrue negative

Classification accuracy is perhaps the simplest metric to use, and it is defined as the number of correct predictions divided by the total number of predictions, multiplied by 100.

Accuracy = (true positive + true negative)/(true positive + true negative + false positive + false negative)

Precision is the ratio of true positive cases to the total positive cases predicted by the model and focuses on type 1 errors.

Precision = true positive/(true positive + false positive)

If the precision value approaches one, it means that a model predicts true positives with high accuracy without producing false positives.

The recall value is the ratio of true positives to the sum of true positives and false negatives and focuses on type 2 errors.

Recall = true positive/(true positive + false negative)

If the recall value approaches one, it indicates that the test is predicting true positives with great accuracy, with a decreased probability of giving false negatives.

The F1 value is calculated using precision and recall values.

F1 = 2/(1/precision + 1/recall)

A high F1 value indicates that precision and recall values are high and there is a good balance between them.

**4. The importance levels of variables:** We analyzed the importance levels of the variables used to predict the development of severe respiratory failure among COVID-19 patients with mild respiratory failure using the SHAP (Shapley additive explanations) method.

## 3. Results

**Figure 2:**
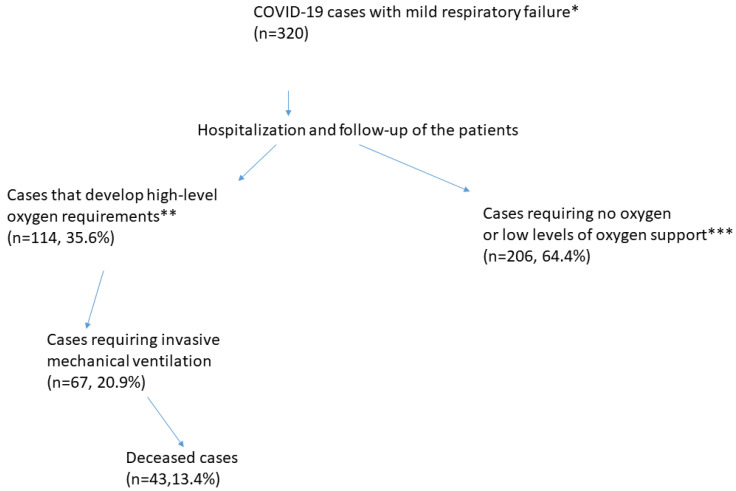
Outcomes of hospitalized COVID-19 patients with mild respiratory failure. *: A resting respiratory rate of ≥22/min, resting oxygen saturation of <94%, saturation that decreases with exercise, and oxygen delivered, at most, via a nasal cannula or mask. **: The use of CPAP, high-flow oxygen support, and mechanical ventilation. ***: The use of a nasal cannula or mask.

This study included 320 patients, 199 males (62.1%) and 121 females (37.8%), with an average age of 54.67 ± 15.82 years. Patients were followed up for a median of 17 (4–65) days. During follow-up, 114 patients (35.6%) experienced severe respiratory failure, 67 (20.9%) required intensive care unit admission, and 43 (13.4%) died (Figure 2).

Table 1 summarizes the patients’ general information, comparing patient groups with and without severe respiratory failure. In the group with severe respiratory failure, the factors of age, Charlson comorbidity index, serum CRP, ferritin and procalcitonin levels, neutrophil count, upper and middle zone CT scores in both lungs, remdesivir usage, and diabetes with end-organ damage rate were higher; lymphocyte counts and the decrease in CRP on the third day were lower than in the group without severe respiratory failure. Violin charts for variables predicting severe respiratory failure are available in the Supplementary Materials, Figure S1.

**Figure 3:**
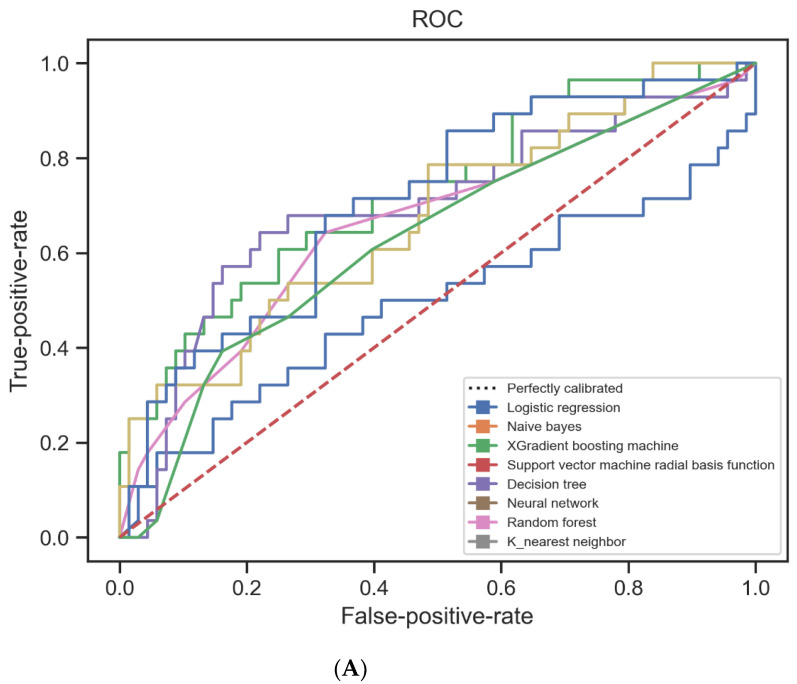

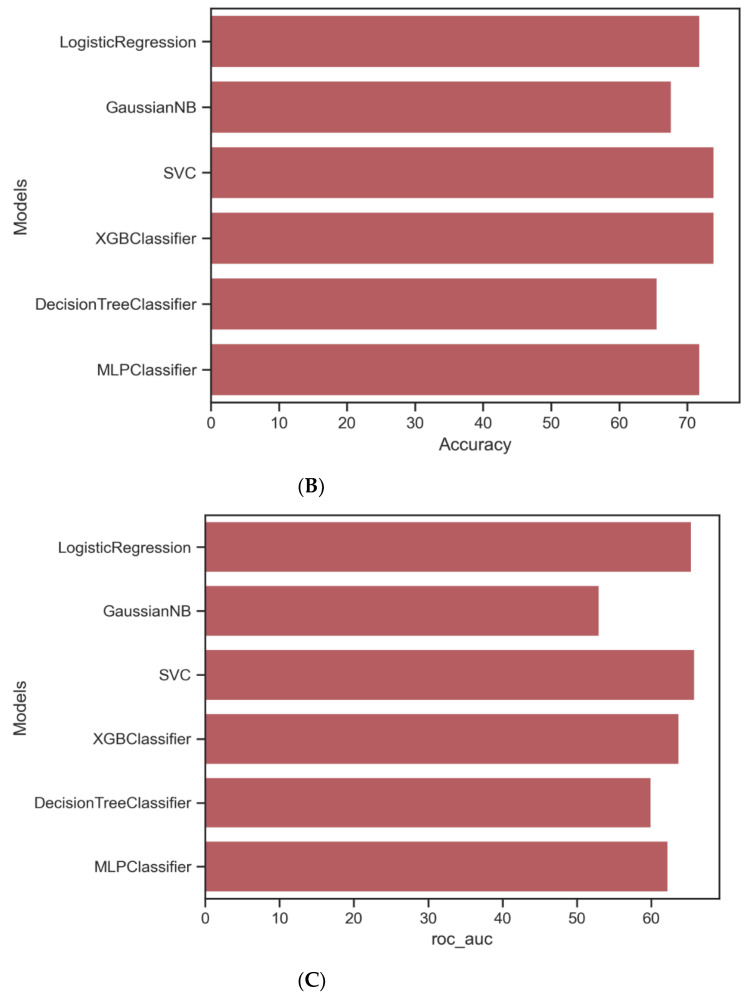
Receiver operating characteristic (ROC) curves (**A**) for the machine learning methods and a comparison of the performance of various machine learning methods used to predict the development of severe respiratory failure (**B**,**C**). The performance metrics, ROC-AUC (area under the receiver operating characteristic curve), and accuracy are plotted for each method (**B**,**C**).

Some machine learning methods were applied to our data to predict the development of severe respiratory failure in COVID-19-infected patients with mild respiratory failure. The performances of these algorithms are compared in Table 2 and Figure 3. The machine learning algorithms with the best accuracy values were XGBoost, support vector machines, k-nearest neighbor, and multi-layer perceptron (the accuracy values of these algorithms were 0.7395, 0.7395, 0.7291, and 0.75, respectively). The precision, recall, and F1 values of these algorithms were similar to and higher than the others (Table 2). When we compared these four methods between themselves, the method with the best accuracy and recall value was multi-layer perceptron (0.75 and 0.75, respectively), while the method with the highest precision and F1 value was k-nearest neighbor (0.89 and 0.79, respectively). K-nearest neighbor had the highest F1 value and thus was the method with the best balance between precision and recall. The algorithm with the best ROC-AUC value was logistic regression, with a value of 0.7274.

The calibration curves of the machine learning methods utilized to predict the development of severe respiratory failure among COVID-19 patients are shown in the Supplementary Materials, Figure S2.

**Figure 4:**
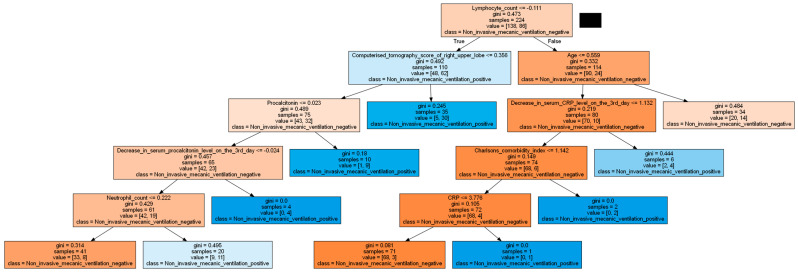
Decision tree for predicting severe respiratory failure.

Next, a decision tree was utilized to predict the development of severe respiratory failure (Figure 4). Initially, lymphocyte counts were evaluated. In cases with low lymphocyte counts, the predictions were based on the right upper lobe computerized tomography score, the decrease in procalcitonin levels on the third day, and neutrophil counts. Conversely, in cases with high lymphocyte counts, the predictions were based on age, the decrease in serum CRP levels on the third day, the Charlson comorbidity index, and the serum CRP level.

**Figure 5:**
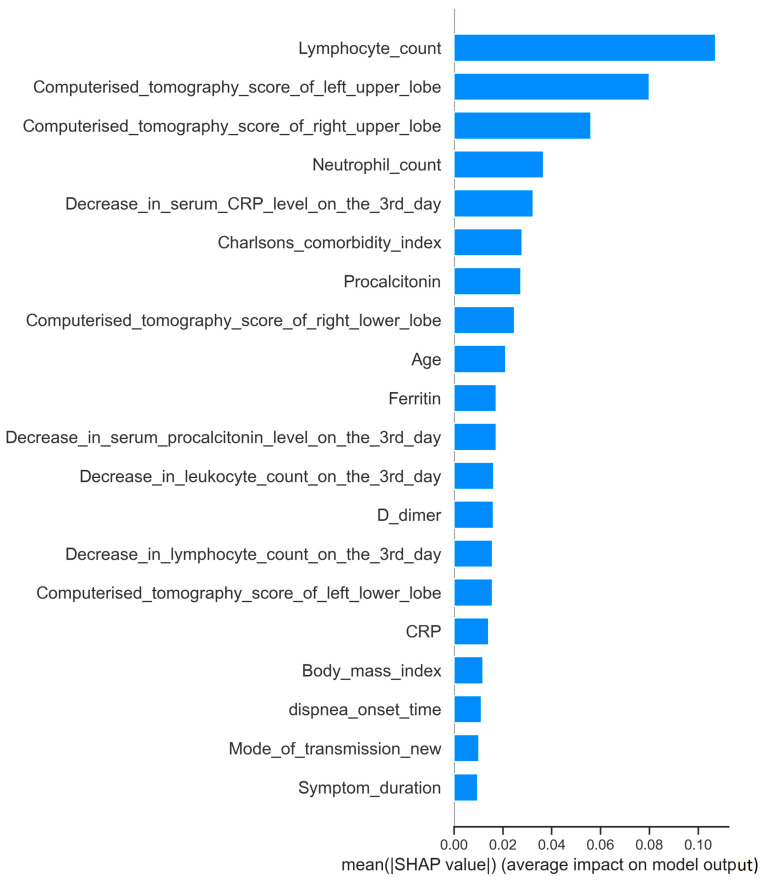
Order of importance of variables for predicting severe respiratory failure. This figure displays the order of importance of variables utilized in predicting the development of advanced respiratory failure among COVID-19 patients with mild respiratory failure. The variables are listed in descending order based on their significance in the prediction model.

**Figure 6:**
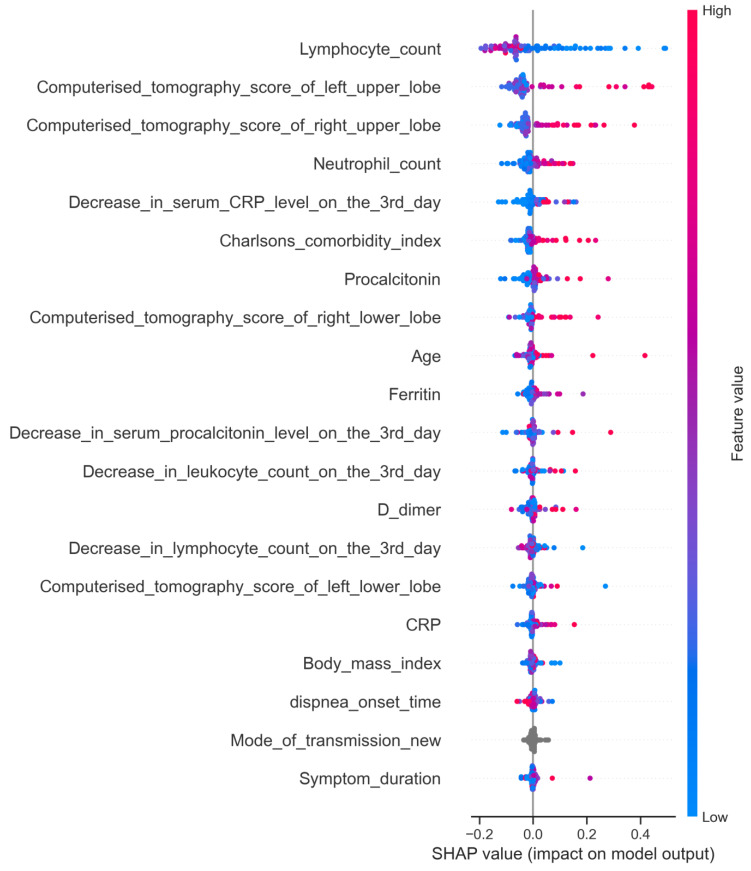
Order of importance of variables for predicting severe respiratory failure among COVID-19 patients. This figure illustrates the order of importance of variables employed in predicting the development of severe respiratory failure among COVID-19 patients. Each dot in the figure represents a data point, with colors indicating actual data values; high values are represented in red, while low values are in blue. The variables are arranged in descending order based on their predictive power, as indicated by the values on the X-axis.

The SHAP method was used to identify the independent variables that contributed most to the prediction of the development of severe respiratory failure in patients with a COVID-19 infection. According to the SHAP test, the variables that could most effectively predict the development of severe respiratory failure were a low lymphocyte count, high left upper and right upper lung zone CT scores, a high neutrophil count, a small decrease in serum CRP levels on the third day of hospitalization, a high Charlson comorbidity index value, and high procalcitonin levels (Figure 5 and Figure 6).

**Figure 7:**
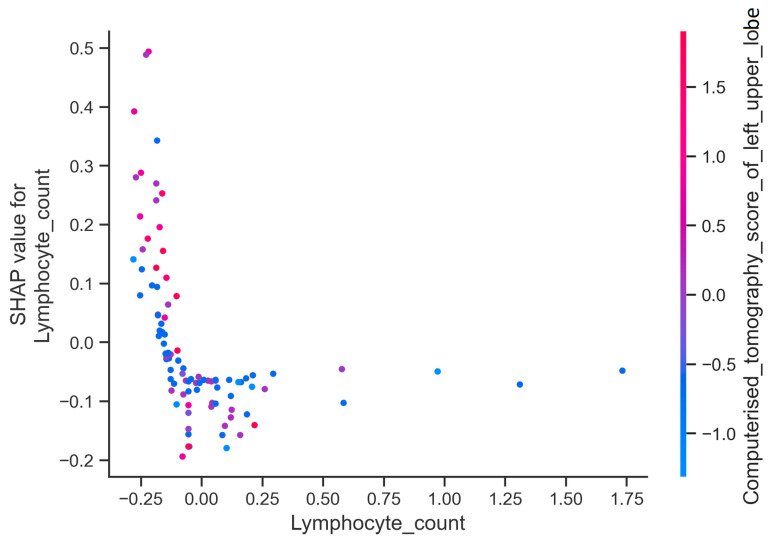
SHAP dependence plot for lymphocyte count and left upper zone computerized tomography score.

The plot shown in Figure 7 demonstrates the relationship between the SHAP value and the values of the lymphocyte count and left upper zone CT score variables, which were identified as the two most significant variables influencing high-level oxygen requirement. In the graph, it is evident that the SHAP value increased as the lymphocyte count decreased and the left upper lobe computerized tomography score increased, suggesting that lower lymphocyte counts and higher left upper zone CT scores are associated with a greater likelihood of severe respiratory failure among COVID-19 patients.

## 4. Discussion

In our study, XGBoost, support vector machines, k-nearest neighbor, and multi-layer perceptron algorithms achieved similar high accuracy, precision, recall, and F1 values. The method with the best accuracy and recall value was multi-layer perceptron, while the method with the highest precision and F1 value was k-nearest neighbor.

The machine learning algorithms we used to detect severe respiratory failure in patients with a COVID-19 infection in our study exhibited a lower performance compared to similar methods in the literature [4,5,6,7,8,9,10,11,12,13,14,15,16,17,18,19,20,21,22,23,24,25]. Comparatively, the ROC-AUC values reported in the literature for similar predictive studies range between 0.669 and 0.99, generally exceeding 0.70 [4,5,6,7,8,9,10,11,12,13,14,15,16,17,18,19,20,21,22,23,24,25]. However, a direct comparison between these studies and ours is challenging due to disparities in the endpoints, predictor variables, sample sizes, and disease severity among patients. Previous studies in the literature typically encompass all COVID-19 cases, both with and without respiratory failure, and often include indicators of respiratory compromise such as oxygen saturation, dyspnea, and respiratory rate [4,5,6,7,8,9,10,11,12,13,14,15]. This comprehensive approach confers an advantage in predicting respiratory failure. In studies in which cases with and without respiratory failure are both included, it is expected that severe respiratory failure will develop in those with a rapid respiratory rate and low oxygen saturation. Furthermore, in one of the studies, the presence of adult respiratory distress syndrome (ARDS) in an X-ray examination was included as one of the predictors of mortality [14]. It is evident that ARDS will significantly predict mortality. Very severely ill patients, such as intensive care patients, patients receiving mechanical ventilation, and those with mild disease without respiratory failure, were not included in our study. Only patients with mild respiratory failure and those followed up in the ward were included in our study, and variables such as ARDS presence in an X-ray examination, oxygen saturation, and respiratory rate indicating respiratory failure were not included in this study. Therefore, our study included a patient group that was more homogeneous than in other studies, highlighting the advantages of our study over other studies. The omission of these strong predictors likely contributed to the lower performance observed in our models and complicated direct comparisons with other studies.

In addition, by utilizing the Shapley method, we found that several factors had a significant influence on the development of severe respiratory failure, including a low lymphocyte count, high left upper and right upper lung zone CT scores, a high neutrophil count, a low decrease in serum CRP levels on the third day of hospitalization, a high Charlson comorbidity index value, and high procalcitonin levels.

In our study, the most important independent variable predicting advanced respiratory failure in COVID-19 cases, found using a SHAP analysis, was the lymphocyte count. In COVID-19 cases, a decrease in peripheral lymphocyte numbers occurs due to various factors, such as TNF-alpha-induced apoptosis, increased peripheral consumption, ACE-2-related cytopathic effects, and interaction with CD147 [40,41,42,43]. Additionally, increased neutrophil numbers can further reduce lymphocyte numbers through a cytotoxic effect [43,44]. Reduced lymphocyte counts have been linked to a poorer prognosis for COVID-19 [44,45]. In our study, we found that low lymphocyte counts and insufficient increases in lymphocyte numbers on the third day were associated with worsening respiratory failure.

Patients with a larger area of lung involvement at onset appeared to have a higher likelihood of developing severe respiratory failure [28]. In our study, in line with the existing literature, individuals who developed severe respiratory failure tended to have higher total computerized tomography scores than those who did not. Studies suggest that COVID-19 often starts in the lower lobes of the lungs and progresses to the upper lobes as the disease becomes more severe, likely due to the larger size and better expansion of the lower lobes [46]. Notably, upper lobe involvement is associated with a more severe disease course, as observed in a few studies [26,27,28]. For instance, one study noted that patients with severe COVID-19 infections admitted to intensive care units often exhibit ground glass opacity, particularly in the right and left upper lobes and the right middle lobe, unlike patients in outpatient settings [26]. In the same study, it was noted that intensive care patients exhibited a higher likelihood of consolidation in the right upper and middle lobes compared to ambulatory COVID-19 patients. In a separate study, the progression of COVID-19 was categorized into four distinct periods spanning different days post-infection (0–9th, 10–14th, 15–20th, and >21st days) [27]. It was observed that, during the initial period (0–9th day), severe cases exhibited more frequent involvement of the left upper and right middle lobes compared to milder cases. However, as the disease advanced into the second and third periods, severe cases demonstrated higher rates of involvement in the right upper and lower lobes, as well as the left upper lobe. These differences were attributed to a reduced blood flow to the upper lobes due to gravitational effects [47,48]. Our study suggests that patients with elevated computed tomography scores in both the upper and lower lung lobes at the outset were more likely to experience disease exacerbations. Moreover, when assessing the influence of certain variables on severe respiratory failure, we observed that the upper zones carried a significantly greater weight compared to the lower zones. Considering that there are few studies in the literature on the effect of upper lobe involvement on disease severity, our study contributes to the literature in this regard. We added a separate value to our study by combining the CT scores of individual lung zones, which are poorly studied, with demographic, clinical, and laboratory values and attempted to predict severe respiratory failure with the help of all these factors.

In one study, it was noted that the activation of the innate immune system and cytokine storms (featuring elevated levels of TNF, IL-6, CXCL10, CCL2, CCL5, and IFN2) play crucial roles in the pathogenesis of COVID-19. Excessive activation of the innate immune system, triggered by viruses and cytokines, leads to a significant rise in neutrophils, serving as a marker for a severe COVID-19 infection [33,34,35]. Elevated neutrophil levels, in turn, activate platelets, release excessive cytokines, and induce epithelial and endothelial damage through a process known as NETosis [49]. In our study, we observed a parallel increase in respiratory failure with elevated neutrophil counts.

C-reactive protein serves as a nonspecific acute-phase protein, whose production is triggered by interleukin-6 in the liver. Elevated serum CRP levels often signal infection, inflammation, or tissue damage. In severe COVID-19 cases, heightened CRP levels reflect the systemic hyperinflammatory state characteristic of severe illness and are associated with a poor prognosis [29,31]. Our study underscores the significance of elevated serum CRP levels and the failure of CRP levels to decrease adequately by the third day; both were closely associated with respiratory deterioration in COVID-19 patients.

Studies have demonstrated that COVID-19 infection tends to be more severe in cases with high Charlson comorbidity index values [50]. Similarly, in our investigation, we found a higher likelihood of respiratory failure in cases with a high Charlson comorbidity index. However, when examining individual comorbidities, these factors ranked lower in predicting respiratory failure development. This could be attributed to the limited number of individual comorbid conditions considered alone. Utilizing a method such as the Charlson comorbidity index, which evaluates comorbidities collectively, highlights these factors’ roles in determining progression to respiratory failure.

It is widely recognized that the severity of COVID-19 escalates with advancing age [51]. In our investigation, we observed a corresponding increase in the risk of respiratory failure with age.

Elevated procalcitonin levels typically signify a bacterial rather than viral infection. It has been proposed that secondary bacterial infections, indicated by high procalcitonin levels, contribute to an unfavorable course of COVID-19 [52]. Moreover, increased procalcitonin levels may be indicative of tissue hypoxia, suggesting that COVID-19 patients experiencing more severe tissue hypoxia are prone to worse outcomes [32]. In our investigation, we found that, if serum procalcitonin levels were elevated initially and failed to decrease adequately by the third day of admission, the likelihood of worsening respiratory failure increased.

Our study had several limitations. Firstly, it was retrospective and conducted at a single hospital, limiting the generalizability of our findings. A larger, multi-center study involving more patients would provide more robust results. However, our hospital, located in Istanbul, a city with a population of approximately 17 million, has a diverse population from a wide region of Türkyie, a factor that helps in capturing a broad spectrum of cases. Another limitation is the relatively small number of cases included in this study. Additionally, we only evaluated a limited set of longitudinally changing variables, such as serum CRP, procalcitonin, and leukocyte counts, while other potentially relevant variables were not assessed. Further studies with a broader scope and a larger sample size are warranted to address these limitations and enhance our understanding of the factors influencing the development of severe respiratory failure in COVID-19 patients.

## 5. Conclusions

The development of severe respiratory failure in patients with COVID-19 with mild respiratory failure could be successfully predicted using machine learning methods, especially logistic regression, XGBoost, multi-layer perceptron, and k-nearest neighbor. In our study, it was seen that the variables that best predicted the development of severe respiratory failure were the presence of underlying diseases, age, neutrophil and lymphocyte counts, the CT score of the upper lung zone, and serum CRP and procalcitonin levels.

## Figures and Tables

**Table 1:** Comparison of demographic characteristics, clinical features, computed tomography scores, and laboratory values of patient groups with or without severe respiratory failure during hospitalization for COVID-19 (0.0: Cases that do not require noninvasive mechanical ventilation; 1.0: Cases that require noninvasive mechanical ventilation).

	Patients with Mild Respiratory Failure (*n* = 206, 64.4%)	Patients with Severe Respiratory Failure (*n* = 114, 35.6%)		*p*-Value
**Age**	53.01 ± 14.29	57.3 ± 17.63		0.025
**Gender (male)**	121 (58.7%)	78 (68.4%)		0.087
**Body mass index**	28.25 (17.99–44.08)	27.76 (17.7–44.9)		0.696
**Previous COVID-19 vaccination**	53 (25.7%)	23 (20.2%)		0.264
**Previous steroid usage**	2 (1%)	4 (3.5%)		0.191
**Immunosuppression**	10 (4.9%)	12 (10.5%)		0.055
**Pregnancy**	8 (3.9%)	3 (2.6%)		0.752
**Charlson comorbidity index**	1 (0–9)	2 (0–9)		0.015
**Mode of transmission**	**Domestic transmission**	97 (47.1%)	48 (42.1%)	0.159
	**Outdoor**	18 (8.7%)	5 (4.4%)	
	**Unknown**	91 (44.1%)	61 (53.5%)	
**Smoking status**	**Smoker**	13 (6.3%)	5 (4.4%)	0.412
	**Nonsmoker**	170 (82.5%)	91 (79.8%)	
	**Ex-smoker**	23 (11.2%)	18 (15.8%)	
**Number of cigarettes smoked (package/year)**		0 (0–120)	0 (0–90)	0.467
**Angiotensin-converting enzyme inhibitor usage**		44 (21.8%)	18 (15.8%)	0.228
**Time from symptom onset to hospital admission**		5 (1–25)	5 (1–30)	0.811
**Time from symptom onset to dyspnea onset**		7 (1–19)	6 (2–14)	0.153
**Symptoms**	**Cough**	170 (82.5%)	91 (79.8%)	0.551
	**Weakness**	49 (23.8%)	34 (29.8%)	0.238
	**High body temperature**	169 (82%)	84 (73%)	0.079
	**Body pain**	133 (64.6%)	58 (50.9%)	0.017
	**Sore throat**	7 (3.4%)	10 (8.8%)	0.04
	**Diarrhea**	21 (10.2%)	5 (4.4%)	0.069
**Remdesivir usage**		4 (1.9%)	12 (10.5%)	0.001
**Favipiravir usage**		98 (47.6%)	62 (54.4%)	0.243
**Comorbidities**	**Liver transplantation**	0 (0%)	2 (1.8%)	0.126
	**Cancer without metastases**	3 (1.5%)	3 (2.6%)	0.365
	**Cancer with metastases**	4 (1.9%)	5 (4.4%)	0.179
	**Heart failure**	5 (2.4%)	6 (5.3%)	0.182
	**Leukemia**	2 (1%)	0 (0%)	0.414
	**Myasthenia gravis**	1 (0.5%)	2 (1.8%)	0.290
	**Lymphoma**	3 (1.5%)	3 (2.6%)	0.365
	**Rheumatoid arthritis**	1 (0.5%)	1 (0.9%)	0.586
	**Aplastic anemia**	1 (0.5%)	0 (0%)	0.644
	**Multiple myeloma**	1 (0.5%)	0 (0%)	0.644
	**Cerebrovascular diseases**	3 (1.5%)	2 (1.8%)	0.585
	**Aortic aneurysm**	1 (0.5%)	0 (0%)	0.644
	**Dementia**	5 (2.4%)	3 (2.6%)	0.589
	**Parkinson’s disease**	3 (1.5%)	1 (0.9%)	0.552
	**Hypertension**	69 (33.5%)	32 (28.1%)	0.317
	**Ischemic heart disease**	13 (6.3%)	13 (11.4%)	0.110
	**Diabetes mellitus**	52 (25.2%)	26 (22.8%)	0.627
	**Diabetes mellitus with end-organ damage**	7 (3.4%)	11 (9.6%)	0.020
	**Chronic obstructive pulmonary disease**	3 (1.5%)	2 (1.8%)	0.585
	**Bronchiectasis**	1 (0.5%)	0 (0%)	0.644
	**Asthma**	5 (2.4%)	5 (4.4%)	0.260
	**Idiopathic pulmonary** **fibrosis**	1 (0.5%)	2 (1.8%)	0.290
	**Severe chronic renal failure**	4 (1.9%)	4 (3.5%)	0.305
	**Mild chronic renal failure**	2 (1%)	4 (3.5%)	0.122
	**Renal transplantation**	3 (1.5%)	2 (1.8%)	0.585
**Laboratory values**	**CRP (mg/L)**	62 (3–283)	86 (3.6–409)	0.003
	**Procalcitonin (ng/mL)**	0.18 (0.01–3.82)	0.21 (0.03–3.62)	0.013
	**D-dimer (ng/mL)**	712 (123–9626)	802 (136–9746)	0.245
	**Ferritin (ng/mL)**	457 (38–20,598)	558 (23–5556)	0.025
	**Leukocyte count (/mm³)**	6300 (720–24,410)	7810 (1860–88,000)	0.008
	**Lymphocyte count (/mm³)**	965 (260–6120)	685 (120–51,000)	0.0001
	**Neutrophil count (/mm³)**	4885 (220–20,380)	6340 (1590–24,190)	0.001
	**Decrease in leukocyte count on the third day (%)**	23 [(−47)–(336)]	34 [(−87)–(339)]	0.896
	**Decrease in neutrophil count on the third day (%)**	36 [(−48)–(509)]	39 [(−84)–(394)]	0.666
	**Decrease in lymphocyte count on the third day (%)**	(−1) [(−73)–(186)]	0 [(−76)–(148)]	0.700
	**Decrease in CRP level on the third day (%)**	(−51) [(−94)–(840)]	(−43) [(−86)–(1682)]	0.002
	**Decrease in procalcitonin level on the third day (%)**	(−39) [(−94)–(207)]	(−28) [(−98)–(68,471)]	0.137
**Computed tomography scores of lung zones**	**Total lungs**	22 (2–84)	28 (2–92)	0.003
	**Right upper zone**	2 (0–12)	4 (0–16)	0.0001
	**Left upper zone**	2 (0–16)	4 (0–16)	0.001
	**Right middle zone**	4 (0–16)	4 (0–16)	0.002
	**Left middle zone**	4 (0–16)	4 (0–16)	0.019
	**Right lower zone**	4 (0–16)	4 (0–16)	0.072
	**Left lower zone**	4 (0–16)	5 (0–16)	0.191

**Table 2:** Accuracy, ROC-AUC, precision, recall, and F1 scores for various machine learning algorithms used in this study to predict the development of high-level oxygen demand, along with their tuning parameters.

Algorithm	Tuning Parameter	Accuracy	ROC-AUC *	Precision	Recall	F1
**Logistic** **regression**	-	0.7187	0.7274	0.72	0.72	0.72
**Naïve Bayes**	-	0.6770	0.5304	0.79	0.68	0.72
**K-nearest neighbor**	K = 12	0.7291	0.5672	0.89	0.73	0.79
**Radial** **basis function support vector machines**	Radial basis function, C = 1, gamma = 0.1	0.7395	0.6586	0.76	0.74	0.75
**Neural** **network**	Activation = relu, alpha = 0.00001, hidden layer size = (10,10,10), solver = sgd	0.7500	0.6764	0.76	0.75	0.76
**XGBoost**	Learning rate = 0.02, maximum depth = 3, n_estimators = 100, subsample = 0.8	0.7395	0.6376	0.79	0.74	0.76
**Classification and regression tree**	Max depth = 5, minimum sample split = 36	0.6525	0.5997	0.65	0.66	0.65
**Random** **forests**	Maximum depth = 10, maximum features = 2, minimum sample split = 10, n_estimators = 1000	0.7187	0.6123	0.77	0.72	0.74

*: Area under the receiver operating characteristic curve.

## Data Availability

We could not share the research data due to ethical reasons regarding sharing patient information.

## Supplementary Materials

The following supporting information can be downloaded at: https://www.mdpi.com/article/10.3390/jcm13237386/s1, Figure S1. Violin charts for variables predicting severe respiratory failure. Figure S1.1. Violin chart for the ‘D-dimer’ variable. Figure S1.2. Violin chart for the ‘CRP’ variable. Figure S1.3. Violin chart for the ‘Body mass index’ variable. Figure S1.4. Violin chart for the ‘Decrease in serum CRP level on the third day’ variable. Figure S1.5. Violin chart for the ‘Charlson comorbidity index’ variable. Figure S1.6. Violin chart for the ‘Ferritin’ variable. Figure S1.7. Violin chart for the ‘Dyspnea onset time’ variable. Figure S1.8. Violin chart for the ‘Decrease in lymphocyte count on the third day’ variable. Figure S1.9. Violin chart for the ‘Decrease in leucocyte count on the third day’ variable. Figure S1.10. Violin chart for the ‘Lymphocyte count’ variable. Figure S1.11. Violin chart for the ‘Procalcitonin’ variable. Figure S1.12. Violin chart for the ‘Neutrophil count’ variable. Figure S1.13. Violin chart for the ‘Computed tomography score of right lower zone of lung’ variable. Figure S1.14. Violin chart for the ‘Symptom duration’ variable. Figure S1.15. Violin chart for the ‘Computed tomography score of right upper zone of lung’ variable. Figure S1.16. Violin chart for the ‘Computed tomography score of left lower zone of lung’ variable. Figure S1.17. Violin chart for the ‘Computed tomography score of left upper zone of lung’ variable. Figure S1.18. Violin chart for the ‘Age’ variable. Figure S1.19. Violin chart for the ‘Decrease in serum procalcitonin level on the third day’ variable. Figure S2. Calibration curves of the machine learning methods utilized to predict the development of severe respiratory failure among COVID-19 patients both before (A) and after calibration (B). These curves assess the agreement between the predicted probabilities and observed outcomes. Ideal calibration is represented by a diagonal line, indicating perfect agreement between the predicted and observed probabilities. Deviations from the diagonal line suggest miscalibration, which can be adjusted through calibration techniques. Figure S2.1. Calibration curves before calibration. Figure S2.2. Calibration curves after calibration.
