# Supplementary material for: Predicting Severe Respiratory Failure in Patients with COVID-19: A Machine Learning Approach
Source: J Clin Med. 2024 Dec 4;13(23):7386. doi: 10.3390/jcm13237386 (PMC11642153; doi:10.3390/jcm13237386)

SUPPLEMENTARY MATERIALS

Figure S1. Violin charts for variables predicting severe respiratory failure.

Figure S1.1. Violin chart for the 'D-dimer' variable

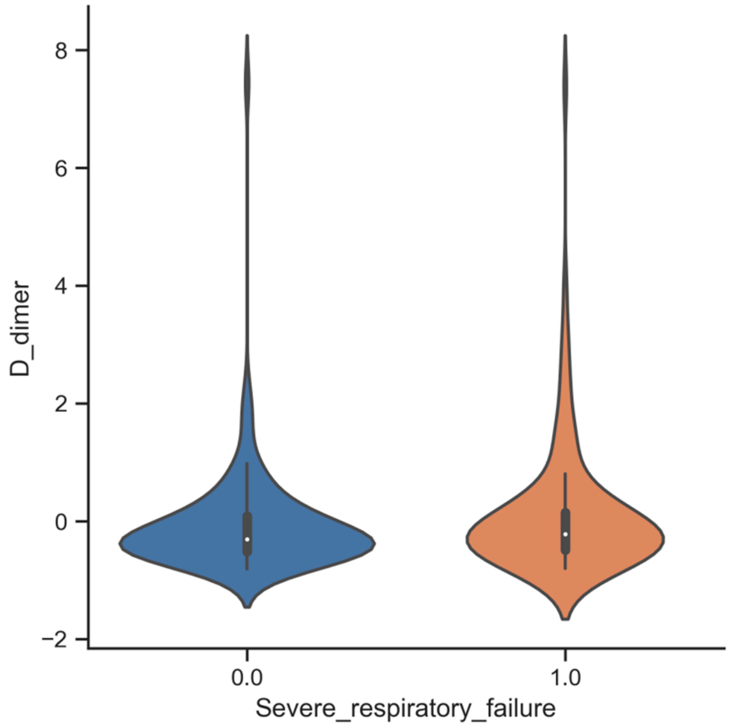

Figure S1.2. Violin chart for the 'CRP' variable

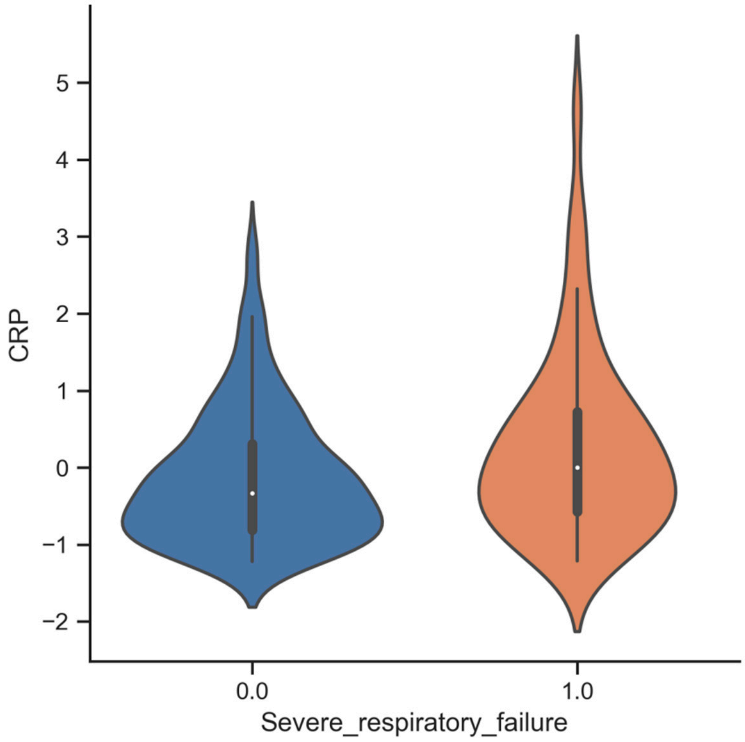

Figure S1.3. Violin chart for the ‘Body mass index’ variable

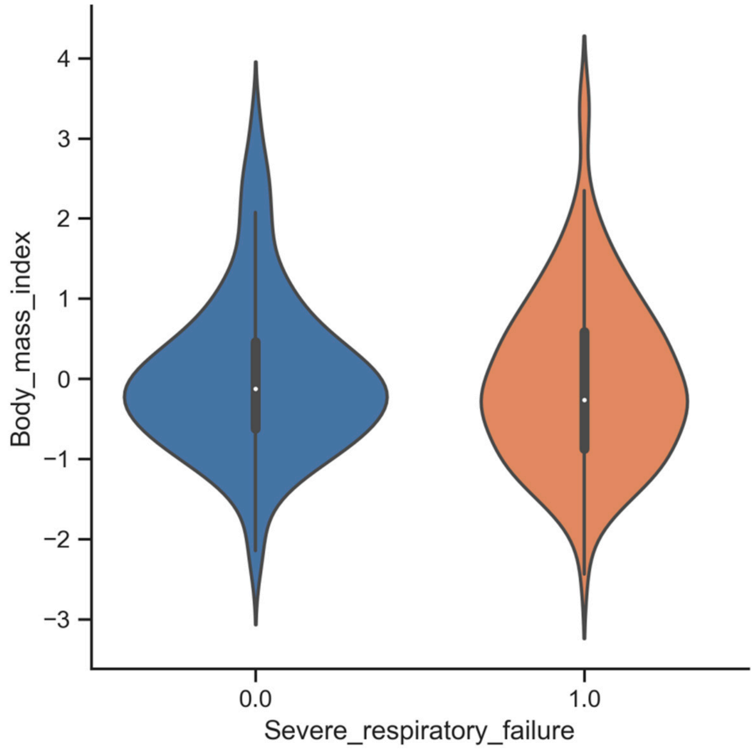

Figure S1.4. Violin chart for the ‘Decrease in serum CRP level on the third day’ variable

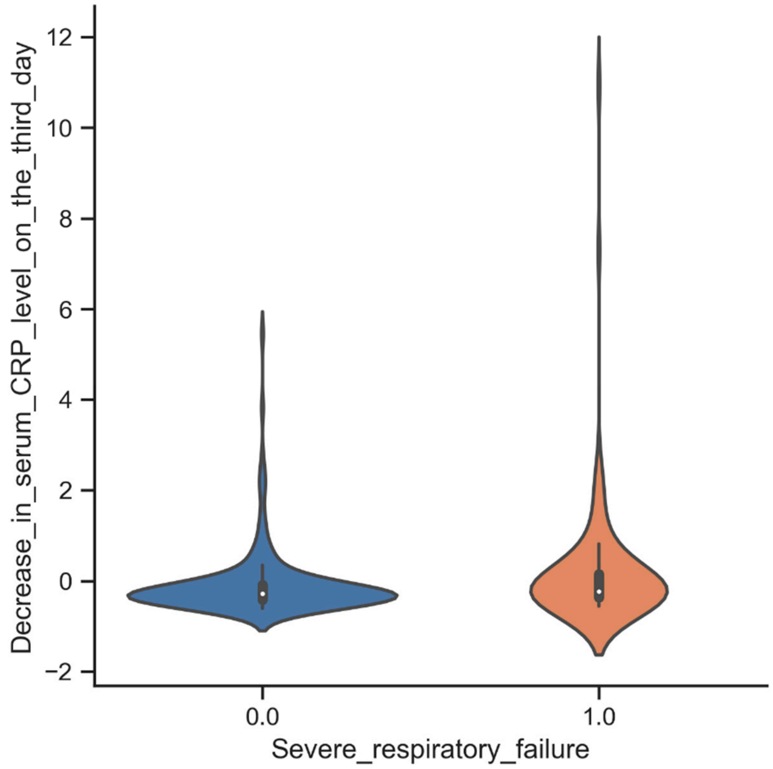

Figure S1.5. Violin chart for the ‘Charlsons comorbidity index’ variable

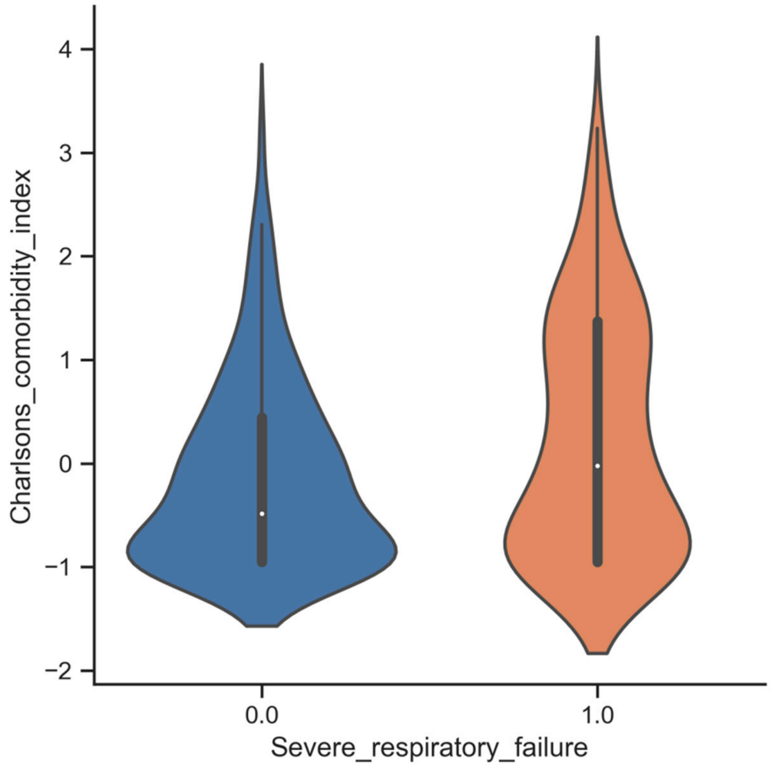

Figure S1.6. Violin chart for the ‘Ferritin’ variable

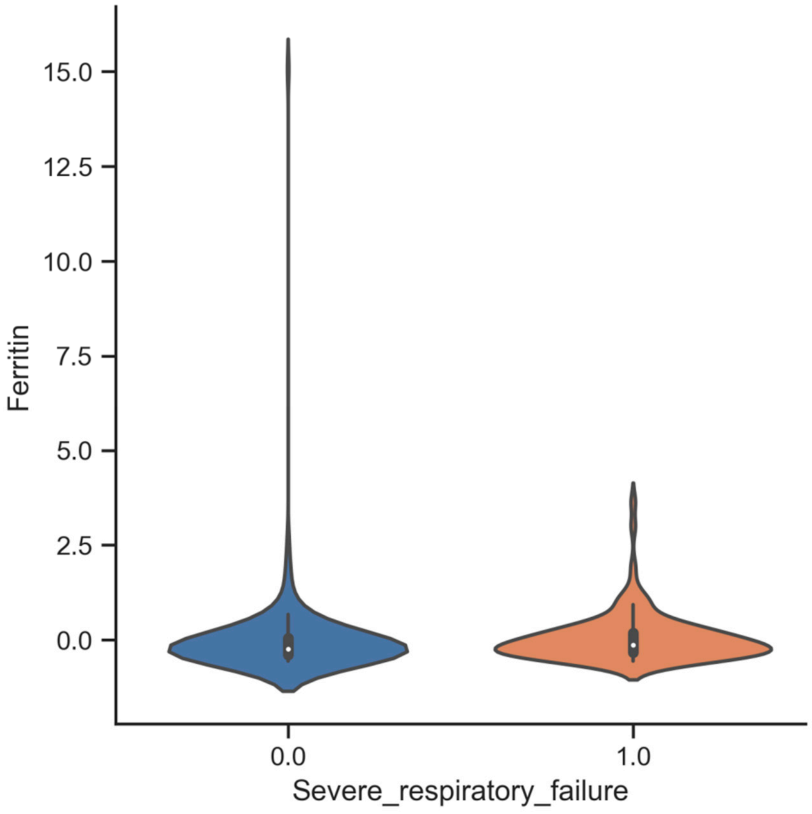

Figure S1.7. Violin chart for the 'Dispne onset time' variable

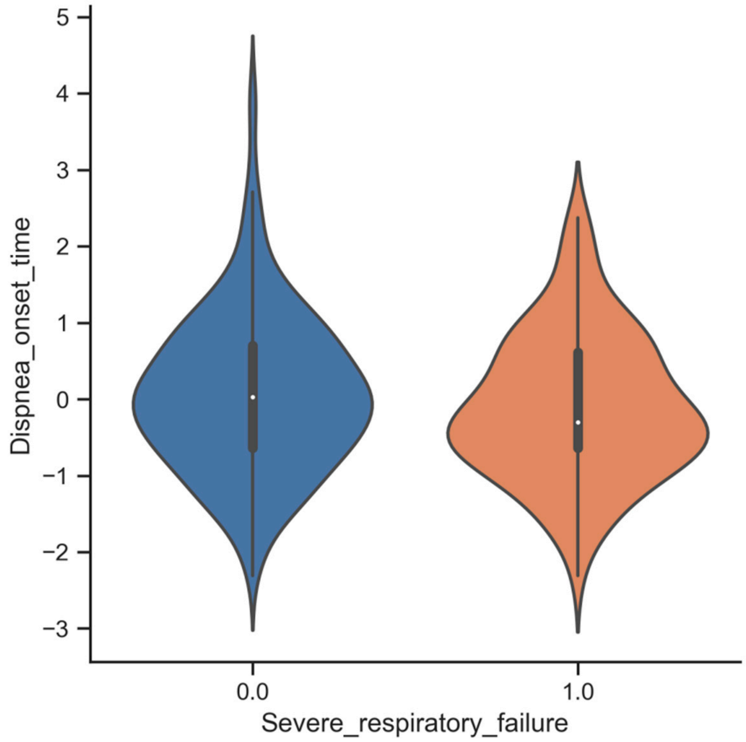

Figure S1.8. Violin chart for the 'Decrease in lymphocyte count on the third day' variable

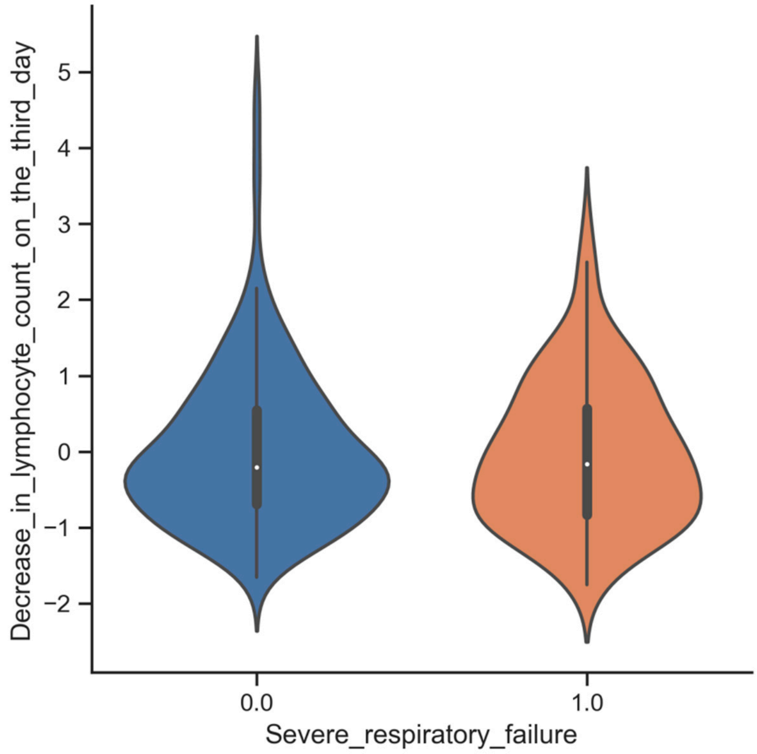

Figure S1.9. Violin chart for the 'Decrease in leucocyte count on the third day' variable

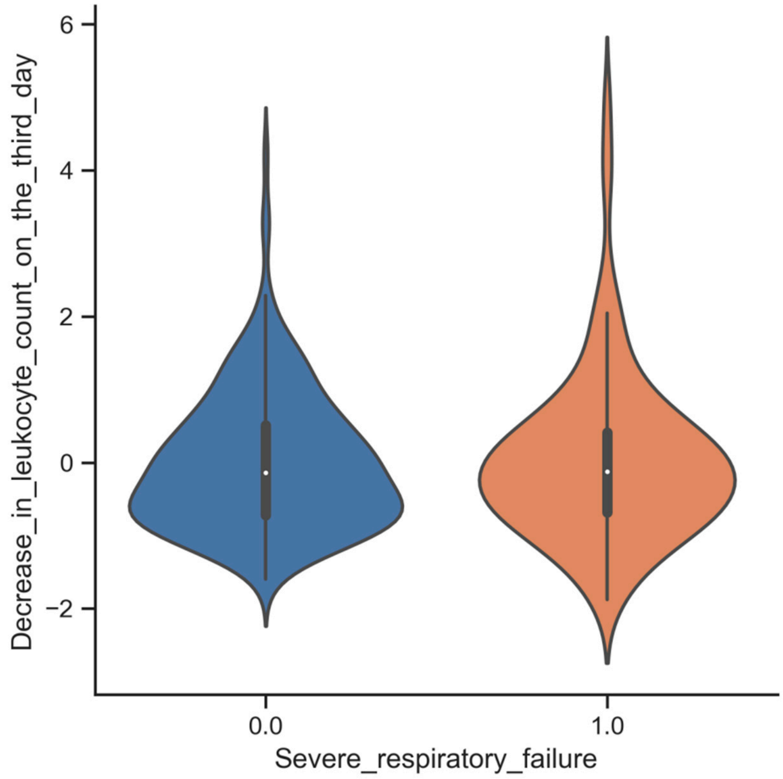

Figure S1.10. Violin chart for the 'Lymphocyte count' variable

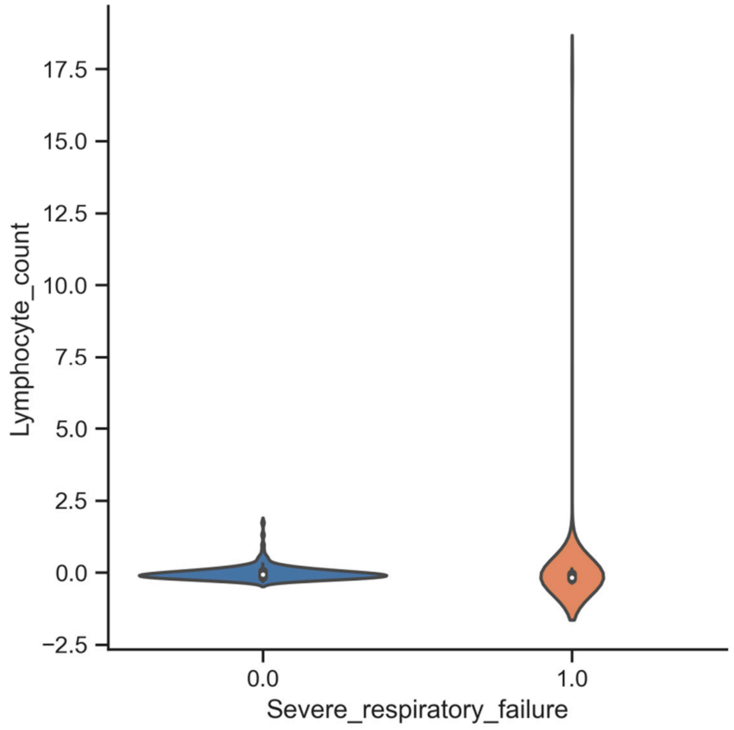

Figure S1.11. Violin chart for the 'Procalcitonin' variable

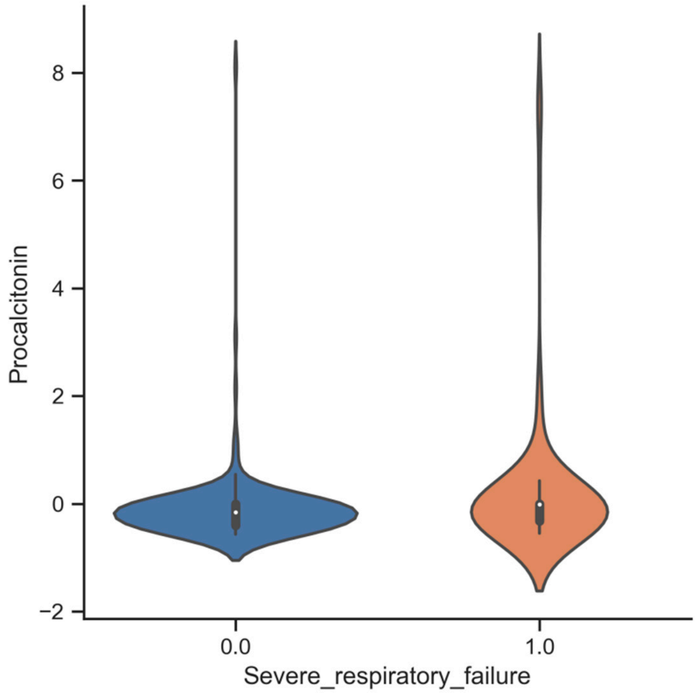

Figure S1.12. Violin chart for the 'Neutrophil count' variable

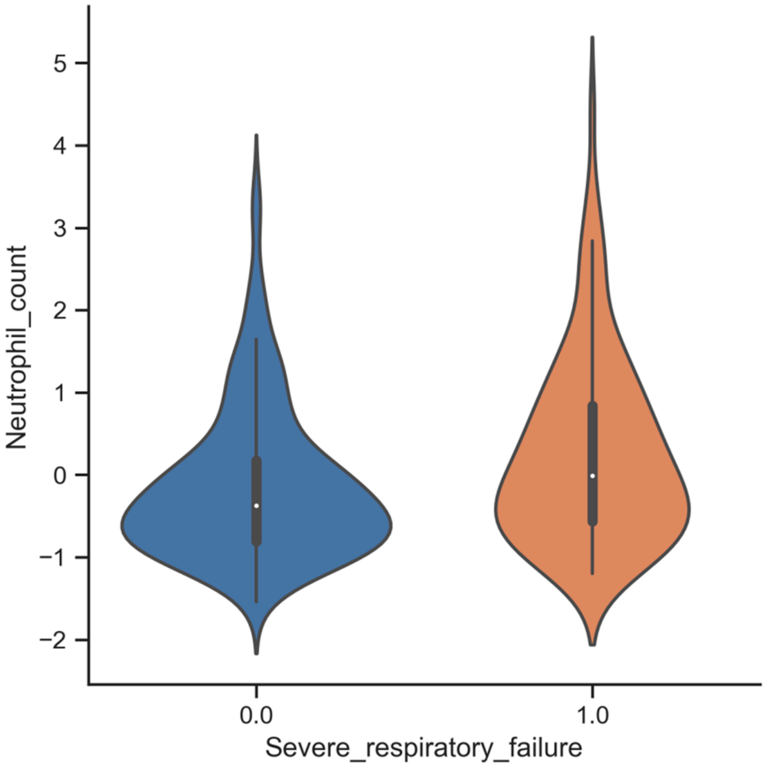

Figure S1.13. Violin chart for the ‘Computed tomography score of right lower zone of lung’ variable

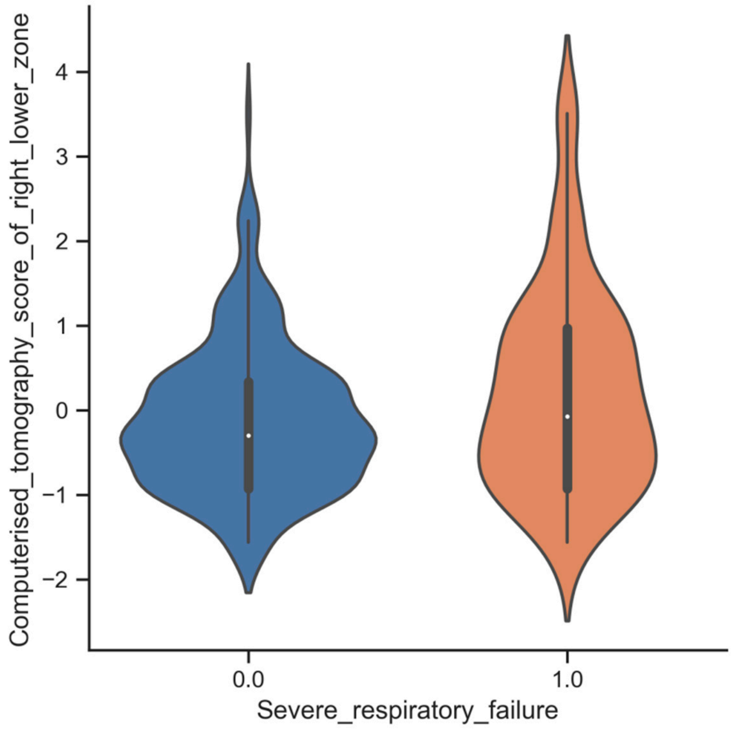

Figure S1.14. Violin chart for the ‘Symptom duration’ variable

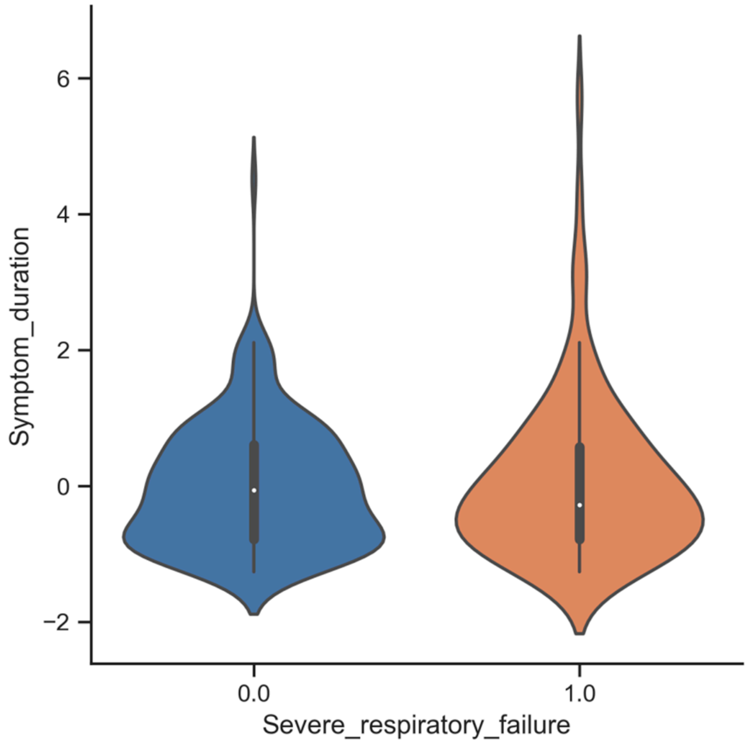

Figure S1.15. Violin chart for the 'Computed tomography score of right upper zone of lung' variable

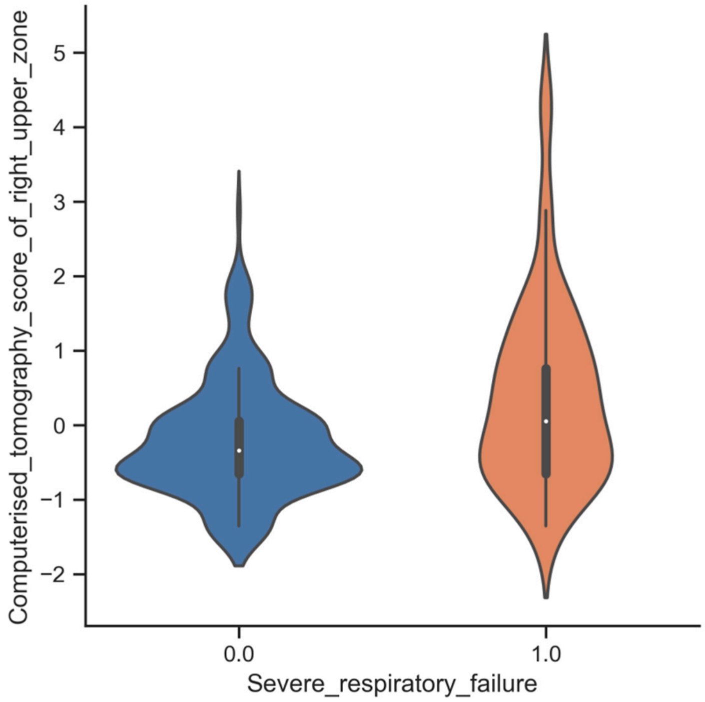

Figure S1.16. Violin chart for the 'Computed tomography score of left lower zone of lung' variable

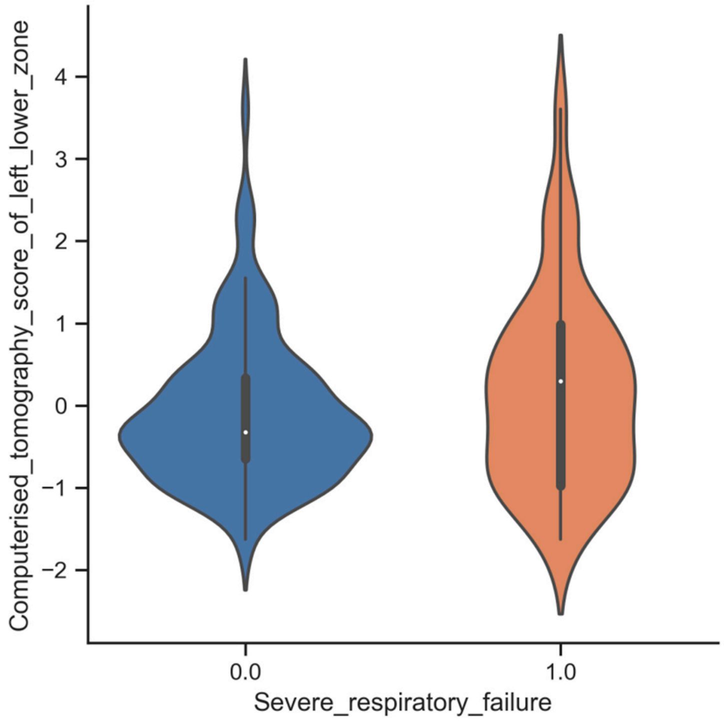

Figure S1.17. Violin chart for the 'Computed tomography score of left upper zone of lung' variable

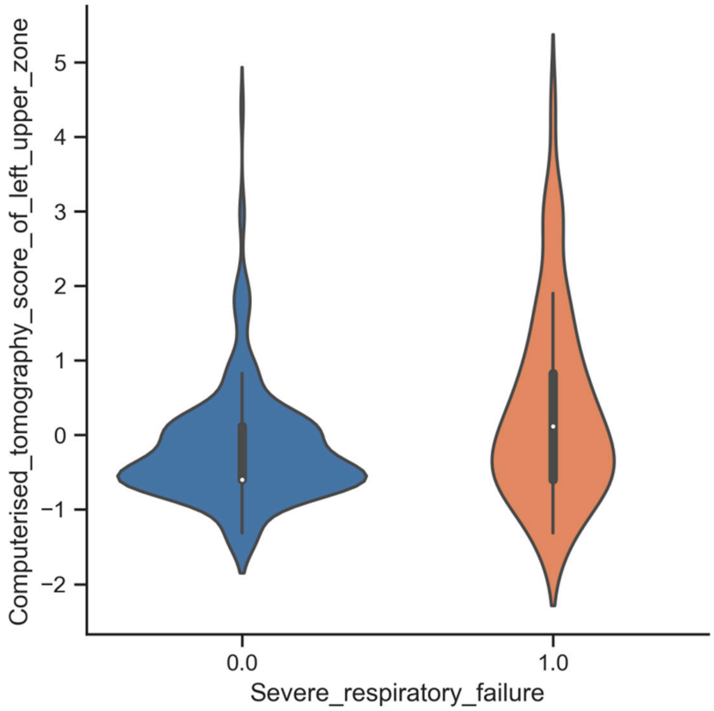

Figure S1.18. Violin chart for the 'Age' variable

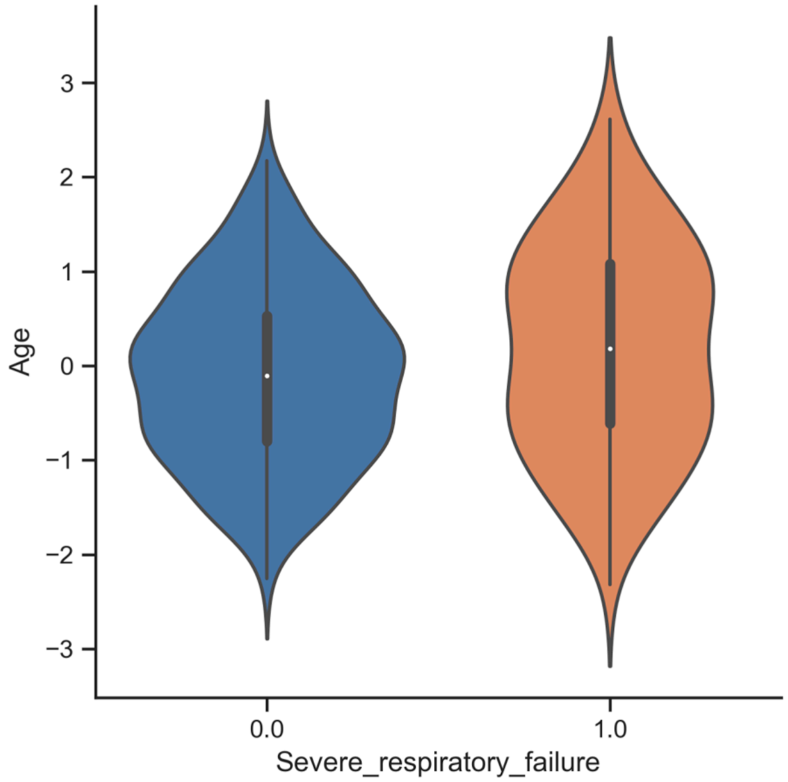

Figure S1.19. Violin chart for the 'Decrease in serum procalcitonin level on the third day' variable

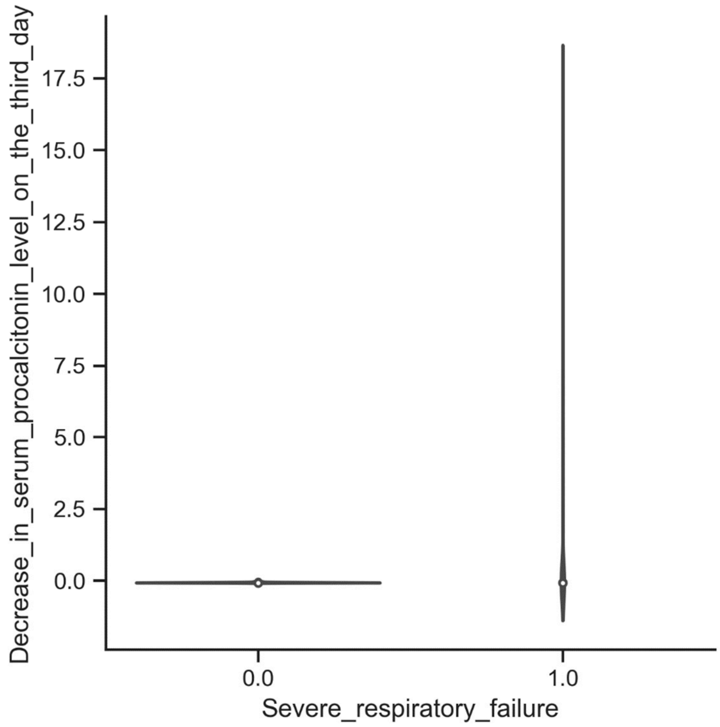

Supplement: Supplementary file 1 [file jcm-13-07386-s001.zip › Supplementary Figure 1.pdf]
