# Supplementary material for: Predicting Severe Respiratory Failure in Patients with COVID-19: A Machine Learning Approach
Source: J Clin Med. 2024 Dec 4;13(23):7386. doi: 10.3390/jcm13237386 (PMC11642153; doi:10.3390/jcm13237386)

Figure S2. Calibration curves of the machine learning methods utilized to predict the development of severe respiratory failure among COVID-19 patients both before (A) and after calibration (B). These curves assess the agreement between the predicted probabilities and observed outcomes. Ideal calibration is represented by a diagonal line, indicating perfect agreement between the predicted and observed probabilities. Deviations from the diagonal line suggest miscalibration, which can be adjusted through calibration techniques.

Figure S2.1. Calibration curves before calibration.

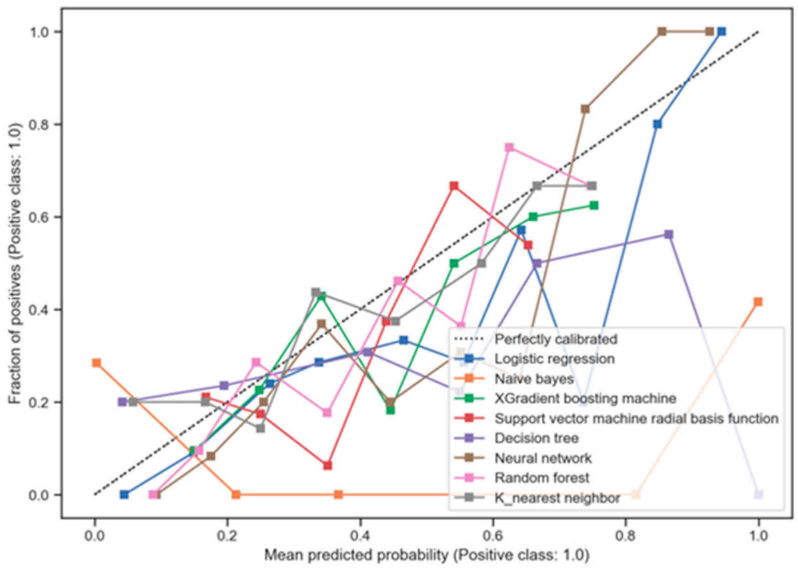

Figure S2.2. Calibration curves after calibration.

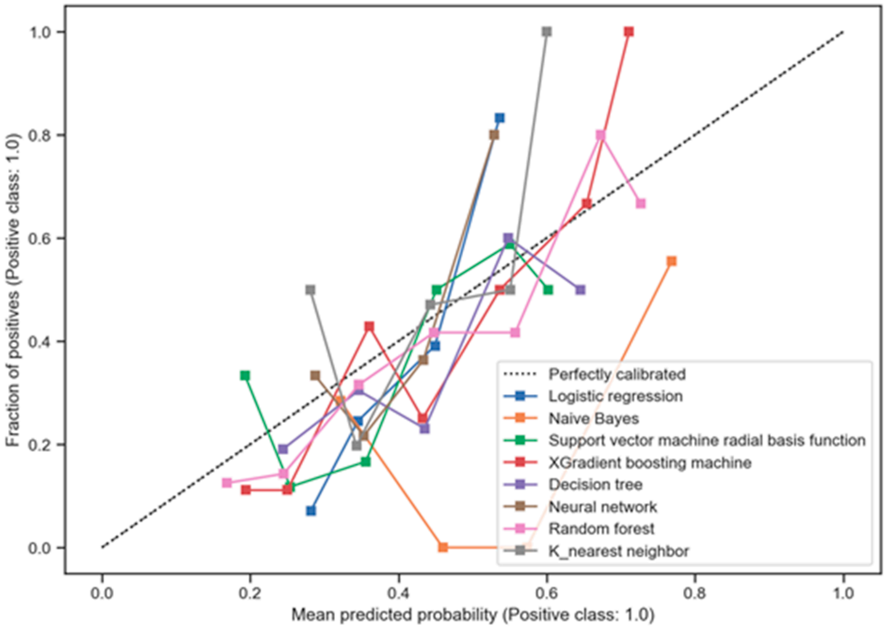

Supplement: Supplementary file 1 [file jcm-13-07386-s001.zip › Supplementary Figure 2.pdf]
